# Environmental Factors Affecting Survival of Immature *Ixodes scapularis* and Implications for Geographical Distribution of Lyme Disease: The Climate/Behavior Hypothesis

**DOI:** 10.1371/journal.pone.0168723

**Published:** 2017-01-11

**Authors:** Howard S. Ginsberg, Marisa Albert, Lixis Acevedo, Megan C. Dyer, Isis M. Arsnoe, Jean I. Tsao, Thomas N. Mather, Roger A. LeBrun

**Affiliations:** 1 USGS Patuxent Wildlife Research Center, Woodward Hall–PSE, University of Rhode Island, Kingston, RI, United States of America; 2 Department of Plant Sciences and Entomology, Woodward Hall, University of Rhode Island, Kingston, RI, United States of America; 3 Department of Fisheries and Wildlife, Michigan State University, East Lansing, MI, United States of America; University of Maryland, College Park, UNITED STATES

## Abstract

Recent reports suggest that host-seeking nymphs in southern populations of *Ixodes scapularis* remain below the leaf litter surface, while northern nymphs seek hosts on leaves and twigs above the litter surface. This behavioral difference potentially results in decreased tick contact with humans in the south, and fewer cases of Lyme disease. We studied whether north-south differences in tick survival patterns might contribute to this phenomenon. Four month old larvae resulting from a cross between Wisconsin males and South Carolina females died faster under southern than under northern conditions in the lab, as has previously been reported for ticks from both northern and southern populations. However, newly-emerged larvae from Rhode Island parents did not differ consistently in mortality under northern and southern conditions, possibly because of their younger age. Survival is lower, and so the north-south survival difference might be greater in older ticks. Larval survival was positively related to larval size (as measured by scutal area), while survival was positively related to larval fat content in some, but not all, trials. The difference in larval survival under northern vs. southern conditions might simply result from faster metabolism under warmer southern conditions leading to shorter life spans. However, ticks consistently died faster under southern than under northern conditions in the laboratory when relative humidity was low (75%), but not under moderate (85%) or high (95%) RH. Therefore, mortality due to desiccation stress is greater under southern than under northern conditions. We hypothesize that mortality resulting from the greater desiccation stress under southern conditions acts as a selective pressure resulting in the evolution of host-seeking behavior in which immatures remain below the leaf litter surface in southern *I*. *scapularis* populations, so as to avoid the desiccating conditions at the surface. If this hypothesis is correct, it has implications for the effect of climate change on the future distribution of Lyme disease.

## Introduction

Lyme borreliosis is the most common vector-borne disease in north temperate regions worldwide [1], with an estimate of about 300,000 cases per year in the United States [2]. The primary vector of Lyme spirochetes in eastern and central North America is the blacklegged tick, *Ixodes scapularis*, which is common in the northeast and the northern midwest, and which ranges throughout the southern states [3, 4]. However, human cases of Lyme disease are primarily reported from the northeast and upper midwest, with relatively few cases reported from the south [2].

Several hypotheses have been proposed to explain this geographical gradient in the distribution of Lyme disease cases. One suggests that increased diversity of vertebrate hosts in the southern U.S. results in dilution of the importance of mice as reservoir hosts in the southern states [5]. Another is the relative abundance of lizards in the south, which results in abundant use of lizards, which are relatively poor as *Borrelia burgdorferi* reservoirs compared to mice, as major hosts for immature ticks [6]. Finally, the increased temperatures in the south might result in changes in tick development time and phenology, which might interfere with the efficient seasonal transmission dynamic of *B*. *burgdorferi* that is characteristic of northern states [7]. Recently, a new phenomenon has been reported that might provide an additional possible explanation for this geographical gradient. Namely, northern ticks apparently differ genetically from southern populations, and this difference is manifested by differences in the host-seeking behavior of nymphal ticks (the major vector stage of Lyme spirochetes) in northern compared to southern sites. The northern nymphs climb to the top of the leaf litter and on twigs to seek hosts, while the southern ticks remain below the leaf litter surface [8]. Therefore, northern ticks frequently attach to humans as hosts, while southern ticks, though present, rarely encounter people.

This behavioral difference was consistently different among populations of ticks that originated from various sites in the northern vs. southern states [9]. Therefore, this difference in behavior might explain the relatively low numbers of *I*. *scapularis* attached to military personnel at southern compared to northern sites [10], as well as the relatively low numbers of nymphal *I*. *scapularis* collected by drag samples in the south compared to the north [11]. The question remains, however: why does this behavioral difference occur between northern and southern populations of *I*. *scapularis*?

A recent study found that *I*. *scapularis* from different locales differed in survival under identical environmental conditions, but that ticks from all populations, regardless of site of origin, survived longer under northern than under southern conditions [12]. This result suggests that southern conditions are less congenial for tick survival than northern conditions. However, southern conditions differ primarily in that temperatures are higher than in the north, so the shorter length of survival might merely result from faster metabolism of these ectothermic animals in warmer environments [13], and not from greater mortality under southern conditions. To understand the significance of this difference in life span under northern compared to southern conditions, it is important to determine whether southern conditions impose additional mortality on the ticks, beyond the physiological effect of faster metabolism under warmer conditions.

In this study we examine the factors that affect survival in immature *I*. *scapularis*, with specific attention to the possible role of potential mortality factors that might differ under northern and southern conditions. To confirm previous results on differences in survival under northern vs. southern conditions, we tested survival of larvae from a cross of northern and southern genotypes. We then assessed the effects of tick body size and fat content on survival of ticks from single locales (to avoid confounding effects of genetic differences between populations), and tested survival under various conditions of temperature and humidity.

## Results

### Survival of north-south crosses

We tested previous results, which reported longer survival under northern than southern conditions regardless of the origin of the ticks [12], by studying survival of larvae from crosses between northern and southern populations. The F_1_ larvae of crosses of male *I*. *scapularis* from Wisconsin with females from South Carolina were observed under northern and southern conditions at high (>90% RH) and moderate (~80–85% RH) relative humidities (Table 1).

**Table 1:** Mean temperatures and relative humidities (±SE) in crosses experiment.

Conditions	Temperature (°C)	N	Relative Humidity (%)	N
**High RH**				
**Northern**	23.2 ±0.22	10	93.2 ±0.55	10
**Southern**	33.2 ±0.25	10	95.3 ±0.72	10
**Moderate RH**				
**Northern**	22.3 ±0.063	16	81.9 ±0.63	16
**Southern**	32.4 ±0.065	16	84.3 ±0.18	16

**Fig 1:**
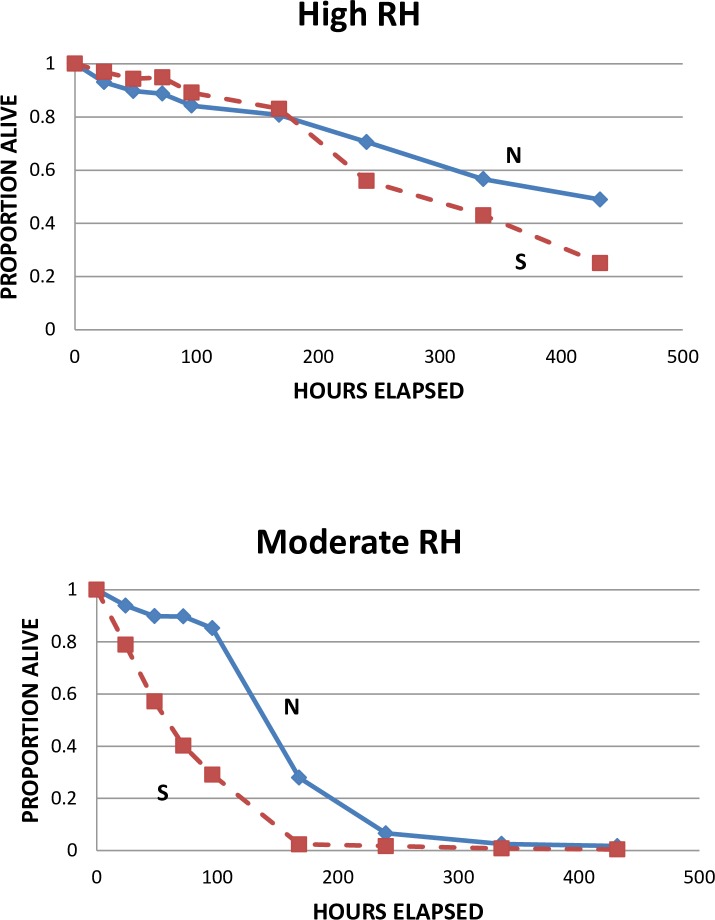
Survival of larvae from crosses of northern fathers (from Wisconsin) with southern mothers (from South Carolina). The data used in this Figure are given in S1 Data.

Under both humidity regimes (Fig 1), the ticks survived longer under northern than under southern conditions (Kolmogorov-Smirnov 2-sample tests, one-tailed, df = 2; high RH, D_max_ = 0.239, χ^2^ = 28.55, p < 0.001; moderate RH, D_max_ = 0.562, χ^2^ = 148.71, p << 0.001). Under both northern and southern conditions, mortality was more rapid under moderate than high RH, with all specimens dead by the end of the experiment under moderate RH, but not under high RH (Fig 1).

### Characteristics of ticks that affect survival

**Fig 2:**
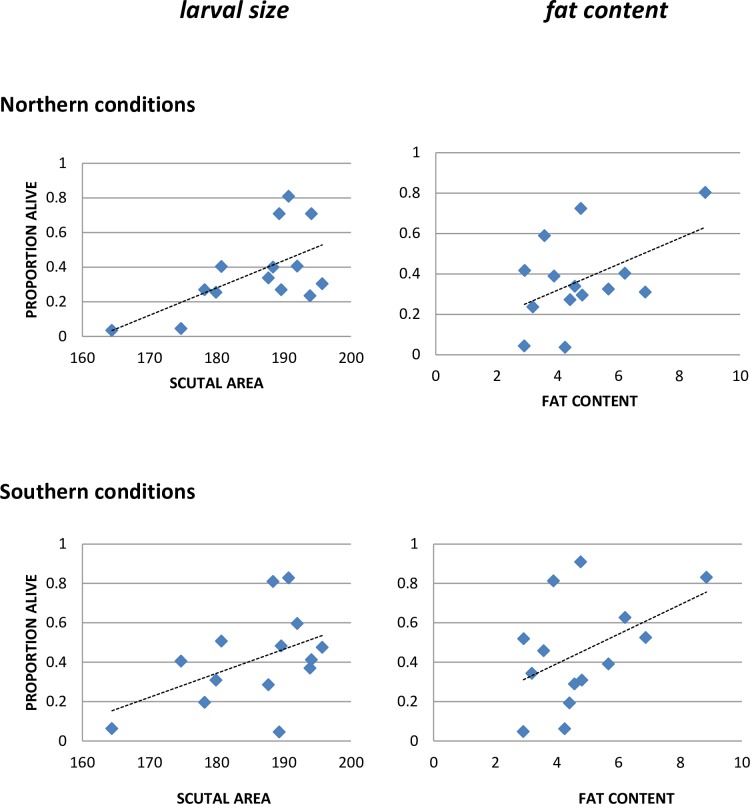
Survival as a function of larval size and fat content– 85% RH (2014 experiment). Vertical axis is mean proportion of ticks in each clutch alive at end of experiment. The data used in this Figure are given in S2 Data.

The larvae used in the crosses experiment (Fig 1) had eclosed >4 months before the experiment began. We tested survival of younger, newly-emerged larvae from adults that had been collected in Rhode Island and fed on rabbits in the laboratory. This provided ticks from just one population, and of the same age, to avoid confounding temporal, geographical, and genetic factors in the estimates of survival. Ticks from 14 clutches (larvae of 14 different females) were held at moderate (~85%) RH under both northern and southern conditions (Table 2). We measured the size and fat content of the ticks from each clutch, and assessed the relationships between these characteristics and survival. Both mean size and fat content of the larvae in the clutch were positively related to larval survival (Fig 2, Table 3). Scutal area and fat content also displayed a significant interaction under both northern and southern conditions, suggesting that the effect of fat content differed for larvae of different sizes.

**Table 2:** Temperature and humidity (±SE) during survival experiment, 2014.

Conditions	Temperature (°C)	N	Relative Humidity (%)	N
**Northern**	22.5 ±0.63	40	85.1 ±0.37	39
**Southern**	32.2 ±0.79	40	82.8 ±0.15	40

**Table 3:** Relationship between larval size and fat content and survival. Entries are *χ*^*2*^ values (*p* in parentheses) for contribution to stepwise logistic regression model.

	Northern conditions	Southern conditions
**scutal area**	70.241 (<0.0001)	139.215 (<0.0001)
**fat content**	29.800 (<0.0001)	21.515 (<0.0001)
**scutal area x fat content interaction**	20.117 (<0.0001)	8.929 (0.0028)

**Fig 3:**
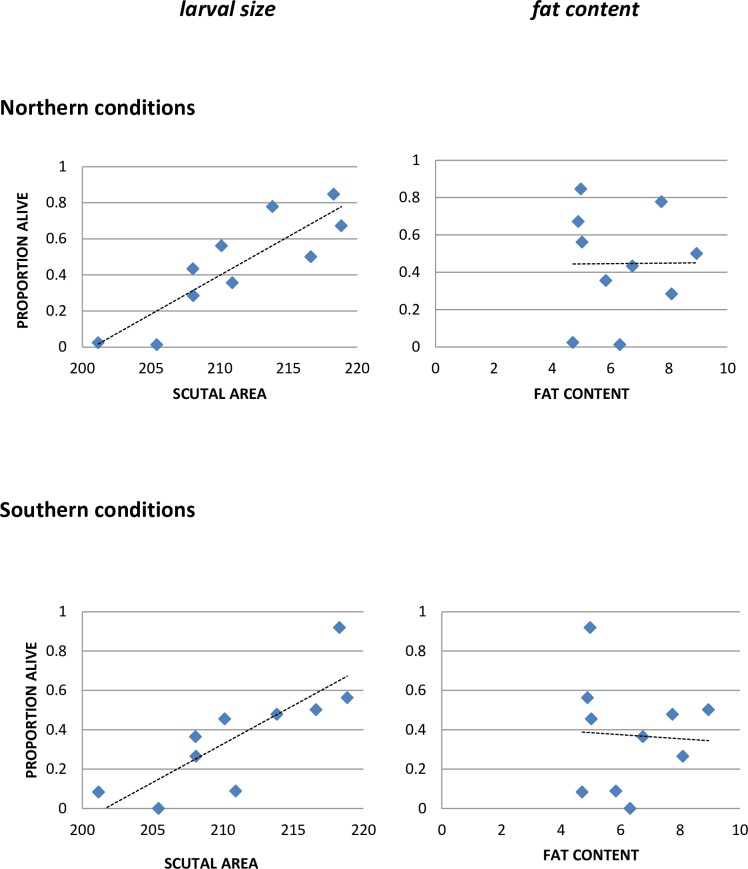
Survival as a function of larval size and fat content– 85% RH (2015 experiment). Vertical axis is mean proportion of ticks in each clutch alive at end of experiment. The data used in this Figure are given in S3 Data.

This trial was repeated in 2015 using 10 clutches, as part of a study on tick survival at different humidities (Table 4). Tick size was again positively related to survival (Fig 3), and this effect seemed stronger in the 2015 experiment than in the 2014 experiment (compare Figs 2 and 3), possibly because this experiment lasted longer (1104 hours in 2015, compared to 768 hours in 2014) and thus exerted greater stress on the ticks. However, fat content did not predict survival in the 2015 experiment at 85% RH.

**Table 4:** Mean temperatures and relative humidities (±SE) in RH-2015 experiment.

Conditions	Temperature (°C)	N	Relative Humidity (%)	N
**High RH**				
**Northern**	21.7 ±0.24	11	95.3 ±0.74	11
**Southern**	31.5 ±0.10	11	96.5 ±0.56	11
**Moderate RH**				
**Northern**	21.4 ±0.060	11	88.3 ±0.36	11
**Southern**	31.1 ±0.063	11	84.5 ±0.28	11
**Low RH**				
**Northern**	21.3 ±0.087	11	75.0 ±0.30	11
**Southern**	31.0 ±0.091	11	76.5 ±0.49	11

We examined the effects of both larval size and fat content on tick survival at all three RH levels, and under both northern and southern conditions, in the 2015 experiment (Table 5). Size was positively and significantly related to survival under both northern and southern conditions, and at all RH levels, except for high RH under northern conditions, where nearly all of the larvae survived. Fat content was generally not significantly related to survival, except under the most stressful circumstance, which was the low RH treatment under southern conditions.

**Table 5:** Relationship between larval size and fat content and survival at different relative humidities and temperatures. Entries are *χ*^*2*^ values (*p* in parentheses) for contribution to stepwise logistic regression model.

approximate RH	75%	85%	95%
**Northern conditions**			
**scutal area**	29.660 (<0.0001)	102.882 (<0.0001)	1.328 (0.249)
**fat content**	0.514 (0.473)	0.094 (0.844)	1.238 (0.266)
**Southern conditions**			
**scutal area**	32.615 (<0.0001)	78.687 (<0.0001)	4.429 (0.035)
**fat content**	4.355 (0.037)	0.011 (0.918)	2.173 (0.140)

### Survival of Rhode Island ticks under northern and southern conditions

**Fig 4:**
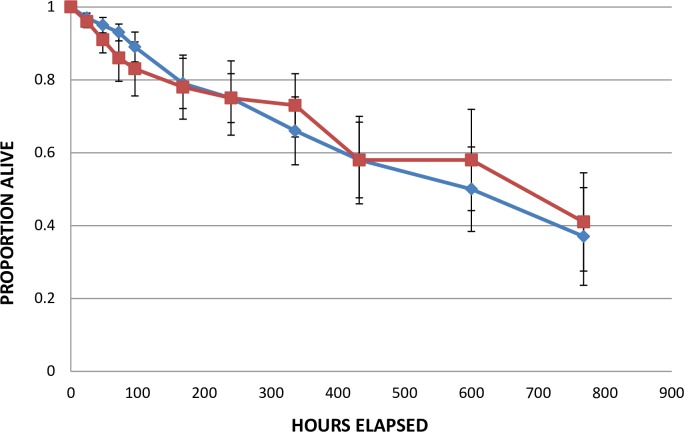
Overall survival patterns of Rhode Island larvae under northern and southern temperature and light:dark conditions at approximately 85% RH. Each data point is proportion alive (±95%CI). The data used in this Figure are given in S4 Data.

Survival of the newly-emerged Rhode Island larvae held at ~85% RH did not differ consistently between northern and southern conditions (Fig 4). One difference between this experiment and the previous experiments (e.g., the crosses experiment, and the experiments reported by Ginsberg et al. 2014 [12], which showed longer survival under northern than under southern conditions), is that these Rhode Island larvae were newly-emerged, while the larvae in the previous experiments had emerged weeks and sometimes months before the experiments began. Survival of older larvae would, of course, be expected to be lower than younger larvae (S1 Fig), so the effects of environmental conditions might be expected to be greater for the older ticks.

**Fig 5:**
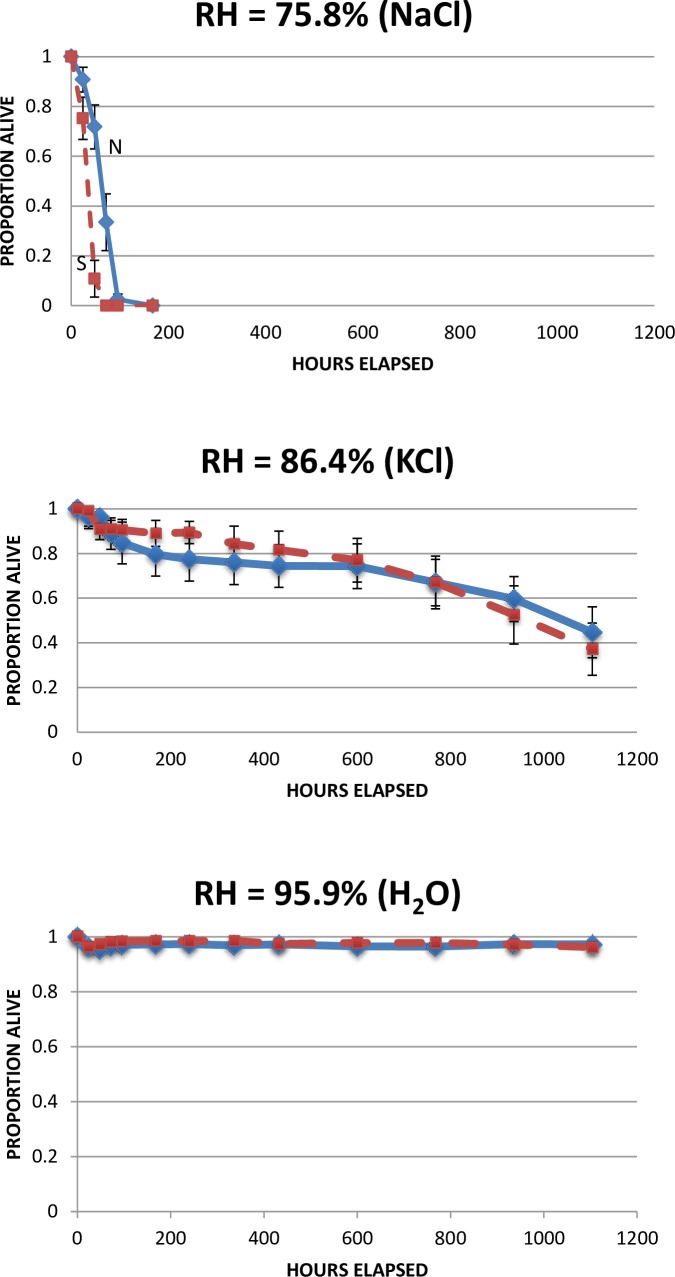
Survival patterns of Rhode Island larvae under northern (blue solid line) and southern (red dashed line) temperature conditions at different relative humidities. Each data point is proportion alive (±95% CI). The data used in this Figure are given in S5 Data.

Our experiment that tested the effect of RH on survival under northern and southern conditions by maintaining newly-emerged Rhode Island larvae under low (~75%), moderate (~85%), and high (~95%) RH conditions (Table 5), showed rapid mortality under low RH, moderate mortality under moderate RH, and virtually all of the larvae survived under high RH for the duration of the experiment (Fig 5). The temperature conditions for this experiment were set to be realistic for northern and southern conditions, by using the mean temperatures during Tick-Adverse Moisture Events (TAMEs) [14] at a northern site (in Cape Cod National Seashore, MA) and a southern site (in the Tall Timbers Research Station near Tallahassee, FL} recorded using HOBO data loggers at leaf litter level. These are the temperatures during TAMEs (days when RH was below 82% for more than 8 consecutive hours), which are predictive of tick mortality [14, 15].

**Fig 6:**
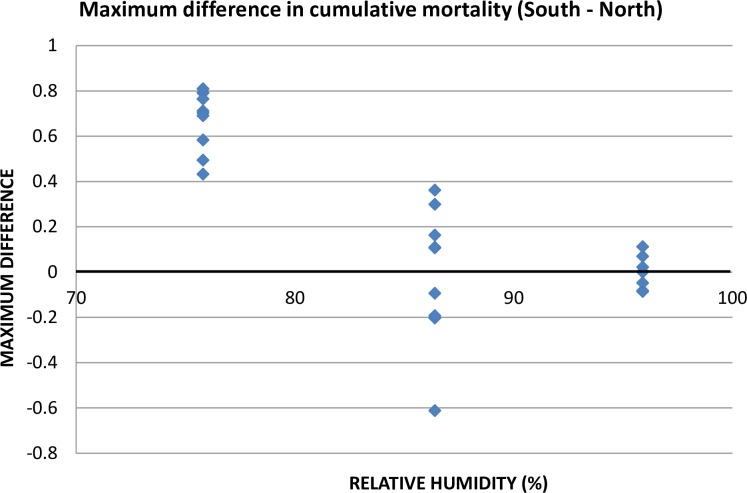
Maximum difference in survival curves (equivalent to D_max_ of Kolmogorov-Smirnov test) of larvae under northern and southern conditions at different relative humidities. Positive values indicate greater survival under northern conditions; negative values indicate greater survival under southern conditions. The data used in this Figure are given in S6 Data.

The differential mortality under southern compared to northern conditions is shown in Fig 6. Each data point is the greatest difference between the cumulative mortality curves for each clutch under each RH treatment. This is equivalent to the D_max_ statistic of the Kolmogorov-Smirnov 2-sample test, except that the signs of the differences were maintained in Fig 6. Proportion mortality of ticks under northern conditions were subtracted from mortality under southern conditions, so a value above zero means that ticks survived longer under northern conditions (in other words, they died more quickly under southern conditions). At high RH, where nearly all ticks survived, the points clustered around zero. At moderate RH there was greater variability, but the points still clustered around zero. At low RH, however, ticks always survived longer under northern than under southern conditions (Fig 6). Therefore, differential mortality under northern and southern conditions was always greatest at low RH, when mortality was consistently greater under southern conditions.

## Discussion

Our results demonstrate that under environmentally-realistic conditions, southern environments exert greater mortality pressure on ticks than is experienced by northern ticks, because of the increased desiccation stress under the warmer southern conditions. We hypothesize that this phenomenon serves as a selective pressure on immature ticks in southern populations of *I*. *scapularis* that favors host-seeking behavior such that the ticks remain below the leaf litter surface, to avoid mortality from desiccation stress at the surface. This would account for the well-known difficulty in collecting immature *I*. *scapularis* by dragging at southern sites [11, 16–18], as well as the very low number of immature *I*. *scapularis* bites experienced by humans in the southeastern U.S. [10, 19, 20]. A person walking through the woods in the north would directly encounter host-seeking nymphal ticks on leaves and twigs at the litter surface, while the same activity in the south would not result in human encounter with immature ticks because the person would remain mostly on top of the leaf litter, and would not encounter the questing ticks below the surface [8]. If this hypothesis is correct, then this climate-related difference in host-seeking behavior has a major influence on human disease risk.

Laboratory conditions often do not accurately reflect conditions faced by organisms in nature, so we took care to use environmentally realistic values of temperature and light-dark cycles in our experiments. Day and nighttime temperatures and light/dark timing in the crosses experiment (Table 1, Fig 1) and the 2014 survival experiment (Table 2, Fig 4) were those at northern and southern field sites during the peaks of larval activity (based on samples of ticks from hosts and on mean temperatures and light/dark times from local weather stations). The 2015 survival experiment looked specifically at the effects of different RH levels on tick survival, so light/dark was set at 15:9 hrs for consistency, but temperatures were based on mean daily temperatures during tick-adverse moisture events (TAMEs) at our field sites (we used temperature and RH data from HOBO data loggers at leaf litter level). TAMEs are days in June when RH is below 82% for >8 hrs, and the number of TAMEs has been found to be inversely related to tick abundance and apparently directly related to mortality of *I*. *scapularis* [14]. Of course, normal daily weather events (such as rain storms) and natural variability in temperature and RH would modify these factors in nature, and our results should be interpreted in view of those possible effects.

The possible roles of temperature and humidity as factors influencing tick behavior are consistent with current knowledge about tick physiological ecology. Water balance is a central physiological factor in tick biology [21, 22]. In the case of *I*. *scapularis*, RH has been shown to affect numerous aspects of tick behavior and ecology, including activity [23, 24], seasonal abundance [14], mortality [15, 25], and vertical movement [26]. Our current report is the first instance, to our knowledge, where the combined effects of temperature and humidity might be implicated as selective pressures affecting geographical trends in tick host-seeking behavior.

We used larvae in these experiments because they respond more rapidly to environmental conditions because of their smaller size and larger surface area to volume ratios than nymphs, so we could get lab results in a timely fashion. Larvae and nymphs are similar in all known ecological patterns (e.g., microhabitat distribution and host-seeking behavior) and both are different from adults [27, 28]. Furthermore, pilot experiments for this study and earlier published experiments showed similar survival patterns for larvae and nymphs, except that nymphs lived longer [12, 25]. Additionally, Berger et al. (2014) presented data suggesting that low RH conditions affect nymphal survival in nature, and we used temperatures in our 2015 survival study that reflected those during low RH conditions at northern and southern field sites. Therefore, it is reasonable to argue that environmental selection pressures on host seeking behavior of larvae would apply to nymphs as well.

An obvious alternative hypothesis for why southern ticks remain below the leaf litter to seek hosts is that lizards are common in the south, and tick questing behavior might be adapted to encounter lizard hosts. However, the only test to date of the preference of southern *I*. *scapularis* nymphs for lizards compared to mice, did not find a consistent difference in host preference in laboratory trials comparing skinks and white laboratory mice [29]. The ticks in these experiments were placed on bridges between caged hosts, while typical questing behavior in *I*. *scapularis* involves little lateral movement [27, 28], with stationary ticks waiting for a host to brush by. It might therefore be informative to repeat these studies using stationary ticks and moving hosts. Furthermore, different lizard species apparently differ in utilization as hosts by ticks. Broad headed skinks and five lined skinks are commonly infested *by I*. *scapularis* in nature, while eastern fence lizards and green anoles are not [6, 30–33], which might partly reflect similarities or differences in the questing microhabitats of the ticks, relative to the foraging microhabitats of the lizards. The potential role of adaptation to important hosts by southern populations of *I*. *scapularis* clearly warrants further study.

The climatic factors that produce increased desiccation stress at the southern leaf litter surface might also affect tick population sizes [10]. Furthermore, related factors, such as longer active seasons in the south, might affect tick phenology, and in turn Lyme transmission dynamics [7]. Study of these factors has been hampered by the relative difficulty of collection free-living nymphal or larval *I*. *scapularis* using standard flag/drag samples in southern states [11]. Our research group is currently evaluating the possible contributions of these factors using nymphs and larvae collected from vertebrate hosts at northern and southern sites. We are also evaluating possible differences in host diversity at northern and southern sites, so as to assess any possible contribution of biodiversity to the north-south gradient in human Lyme disease.

It is important to note that this apparent north-south difference in vulnerability to desiccation at the litter surface might differ for different tick species. For example, nymphal *Amblyomma americanum* survive longer in open habitats than *I*. *scapularis* [34], presumably because they are less vulnerable to desiccation [35]. Nymphal *A*. *americanum* readily seek hosts high up in the vegetation well above the leaf litter.

The average size of larvae in a clutch was generally predictive of clutch longevity (Figs 2 and 3, Tables 3 and 4). Larval fat content was also predictive in the 2014 experiment, but not in the 2015 experiment, except under stressful conditions (high temperatures and low RH). The larvae used in these experiments were newly emerged, and larger size would be expected to confer some resistance to desiccation stress because of lower surface area to volume ratios. Fat content might well play a more important role in older ticks, because fat stores decline in the older individuals. However, the reasons for different results in these two experiments are not clear. Both mean scutal area and fat content were higher in the 2015 larvae [36], but the implications of that difference are not obvious. The larvae in the 2014 experiment were from spring-collected adults, while those in the 2015 experiment were from fall-collected, presumably younger adults, and the adults were from different sites, so age-related or genetic differences among the adults might have affected the role of fat content in larval survival. Further research is needed to clarify the reasons for these different results. Engorgement weights of female ticks can affect the number of eggs they lay, and the average size of their larvae [36, 37], and can thus potentially affect the number of offspring that live long enough to find a host. Female ticks feeding on different hosts have been shown to differ in engorgement weight [38, 39], so host choice can have implications for the probability of offspring survival, and thus for fitness.

Our hypothesis has potentially interesting implications for the effects of climate change on the distribution of Lyme disease. Several modeling studies, as well as recent evidence, suggest that a generally warming climate will result in northward spread of the northern limit of *I*. *scapularis* populations, and of Lyme disease as a public health problem [40–43]. However, effects of climate change on the southern portion of the range are less clear, because there are two mechanisms at work. One is the expansion of the range of *I*. *scapularis*, which apparently includes southward as well as northward expansion [4]. For example, the range of Lyme disease cases in recent decades has extended southward along the Blue Ridge mountains in Virginia [44], which suggests southward range expansion of northern genotype ticks. The other mechanism, however, is the potential change in environmental conditions (resulting from climate change) that foster transmission of Lyme disease to humans. Northern populations of *I*. *scapularis* apparently do not differ from southern populations in vector competence for *Borrelia burgdorferi* [45]. However, host associations and questing behavior apparently differ substantially between northern and southern populations. Northward expansion of dense populations of skinks, or selective pressure from warmer temperatures resulting in southern-style host-seeking behavior, might eventually lower the incidence of Lyme disease in the mid-Atlantic states, especially in areas at low altitudes, such as the Chesapeake Bay area. Effects of climate on vector behavior are relatively poorly understood, and can potentially influence the epidemiology of vector-borne pathogens. The environmental reasons for the host-seeking behavior of southern *I*. *scapularis* might therefore be critical to predictions of future geographical trends in the epidemiology of Lyme disease.

## Materials and Methods

### Crosses experiment

Larvae from four clutches (from 4 different females) were assigned at random to 8 humid chambers. The larvae were placed in 3-dram plastic snap-cap vials with screened tops in the humid chambers (S2 Fig), which were placed in Percival I-36LL environmental chambers (Percival Scientific, Perry, IA). Four were held under northern conditions (L;D 14.5:9.5 hrs, temperature 23.3°C day, 16.7°C night), and 4 under southern conditions (L:D 14.2:19.8 hrs, temperature 32.2°C day, 18.3°C night). Two vials from each clutch were placed at random into each humid chamber (total of 64 vials, mean = 15.4 larvae/vial). These conditions were based on mean conditions during the peaks of larval activity in the northeast (Chatham, MA on 1 August) and southeast (Aiken, SC on 15 June). Two humid chambers in each Percival were maintained at approximately 85% RH using a saturated salt solution of KCl [44], and two at approximately 95% RH using deionized water. Temperature and RH were measured using Temp/RH Pens (Traceable ISO 17025 Calibrated Humidity/Temperature Pens, Control Co., Friendswood, TX) placed within the humid chambers. The vials were checked periodically for tick mortality, using the methods of Ginsberg et al. 2014 [12].

The larvae were obtained from four crosses of southern female *I*. *scapularis*, which were the F_1_ of engorged females collected from deer in South Carolina, with northern males, which were the F_1_ of engorged females collected from deer in Wisconsin. The adults were mated on New Zealand White rabbits (*Oryctolagus cuniculus*), as detailed by Arsnoe 2015 [9]. The crosses and tick feeding were performed at Michigan State University (MSU), and the protocol was approved by the MSU Institutional Animal Care and Use Committee (protocol 06-12-103-00). Egg hatch began on 1–10 February, and the tick survival experiment began at the University of Rhode Island on 23 June 2014 (average period between the onset of egg hatch and beginning of the survival experiment was 136.5 days).

### Rhode Island tick survival experiments

Larvae used in the 2014 experiment were the F_1_ of adults collected at 2 sites along Middle Bridge Road in South Kingstown, RI, in April, and fed on a rabbit in the laboratory. Protocols for animal care and tick feeding for this study were approved by the University of Rhode Island Institutional Animal Care and Use Committee (protocol AN08-04-017). After oviposition and egg hatch, larvae were held at 22.4°C and at >95% RH until the experiment began, soon after eclosion was complete. The experiment started on 14 July, with clutches from 14 females, and lasted 768 hours. Larvae were placed into snap-cap vials with screen tops, as in the crosses experiment, and assigned randomly to treatments. Two vials with larvae from each clutch were placed into each of 8 humid chambers (total of 224 vials, mean = 12.1 larvae/vial), with 4 chambers held under northern conditions (L:D 14.5:9.5 hrs, temperature 23.3°C day and 16.7°C night) and 4 held under southern conditions (L:D 14.2:9.8 hrs, temperature 32.2°C day, 18.3°C night) as in the crosses experiment. RH was maintained at ~85% using saturated KCl solutions in the humid chambers [46].

The 2015 experiment was designed to compare survival under cooler northern conditions with warmer southern conditions at different RH levels. The larvae were the F_1_ of 10 females that had been collected at 2 sites along South Road in South Kingstown, RI, in October 2014, and fed on a new rabbit in the laboratory. The larvae were placed in the experimental vials on 6 April 2015, soon after eclosion was complete, and the experiment lasted 1104 hrs. Northern conditions reflected daytime temperatures during tick-adverse moisture events (TAMEs) recorded during June 2011 at the Eastham sampling array at Cape Cod National Seashore, MA, using HOBO data loggers (Onset Computer Corp, Bourne, MA) at leaf litter level [14, 47], and southern conditions were the daytime temperatures during TAMEs at the NB 66 array at Tall Timbers Research Station near Tallahassee, FL. Northern temperature conditions in the Percival environmental chamber was set at 21.3°C day and 16.7°C night, and southern temperature conditions were 31.9°C day and 18.3°C night, with both treatments at L:D 15:9 hrs. Three vials from each clutch were placed at random into 6 humid chambers (= 180 vials, mean = 15.0 larvae/vial), with one humid chamber at each RH level placed in each of the Percivals (the Percivals were switched in terms of northern vs. southern conditions for this experiment to avoid any incubator effect). The RH levels were maintained in the appropriate humid chambers at ~95% using deionized water, ~85% using a saturated KCl solution, and ~75% using a saturated NaCl solution (Table 4).

### Measurements of tick size and fat content

Tick size was measured independently of fat content using scutal area as a surrogate for larval size. We used scutal area as a measure of tick size because it is independent of weight, which is used in the determination of fat content. Scutal size (measured as length) is positively related to unfed weight in *Rhipicephalus appendiculatus* [48]. After the experiment, larvae were preserved in 95% ethanol until measurement (details given by Ginsberg et al [36]). Briefly, one larva was selected at random from each vial, length and width of the scutum was measured using a Wild M3Z dissecting microscope (Leica Microsystems, Wetzlar, Germany) with an eyepiece reticle (1 reticle unit = 0.017 mm) and area was approximated as an ellipse.

Fat content of larvae was measured using the Lee and Volson colorimetric method [36], in which fat was extracted from pools of 100 larvae using chloroform-methanol, and fat content of each pool was determined using a vanillin-phosphoric acid reagent and comparing absorbance to a standard curve created using corn oil dilutions. Fat content was calculated per unit tick weight for each pool (see [36] for details).

### Statistical analyses

Cumulative mortality curves were compared in the crosses experiment using Kolmogorov-Smirnov two-sample tests, one-tailed [49], to determine whether mortality was greater under southern than under northern conditions. Mortality in the different treatments of temperature (northern vs. southern) and RH in the subsequent experiments were analyzed using logistic regression to compare the number alive vs. dead in each treatment at the conclusion of each experiment. Logistic regressions were performed using SAS, version 9.3 (Cary NC), LOGISTIC procedure, with stepwise selection of size, fat content, and interaction terms.

## Supporting Information

S1 Data Spreadsheet containing data displayed in Fig 1. (CSV)

S2 Data Spreadsheet containing data displayed in Fig 2, Table 3. (CSV)

S3 Data Spreadsheet containing data displayed in Fig 3, Table 5. (CSV)

S4 Data Spreadsheet containing data displayed in Fig 4. (CSV)

S5 Data Spreadsheet containing data displayed in Fig 5. (CSV)

S6 Data Spreadsheet containing data displayed in Fig 6. (CSV)

S1 Fig Survival patterns of larval *I. scapularis* of different ages, from adults originating from Massachusetts, Wisconsin, and Michigan. Mean proportion of larvae alive at end of experiments (672 hrs) for larvae from same cohorts that were tested twice at different ages. These were larvae whose survival was compared with those of southern populations under northern and southern conditions [12]. Larvae from northern and southern populations often did not emerge simultaneously, so larvae from MA, WI, and MI were tested twice, once when the larvae from the southern sites were tested, and later when the larvae from these northern sites were the same ages as the larvae from the southern sites (those comparisons were reported by Ginsberg et al. 2014 [12]). The results shown here from MA and WI larvae indicate survival at ~95% RH, while those from MI were at ~85% RH. Approximate mean ages of the ticks were: MA and WI, young 63 days, old 108 days; MI, young 22 days, old 77 days.
(TIF)

S2 Fig Experimental vials in humid chamber. (TIF)
