# Supplementary figures and images for: Environmental Factors Affecting Survival of Immature Ixodes scapularis and Implications for Geographical Distribution of Lyme Disease: The Climate/Behavior Hypothesis
Source: PLoS One. 2017 Jan 11;12(1):e0168723. doi: 10.1371/journal.pone.0168723 (PMC5226345; doi:10.1371/journal.pone.0168723)

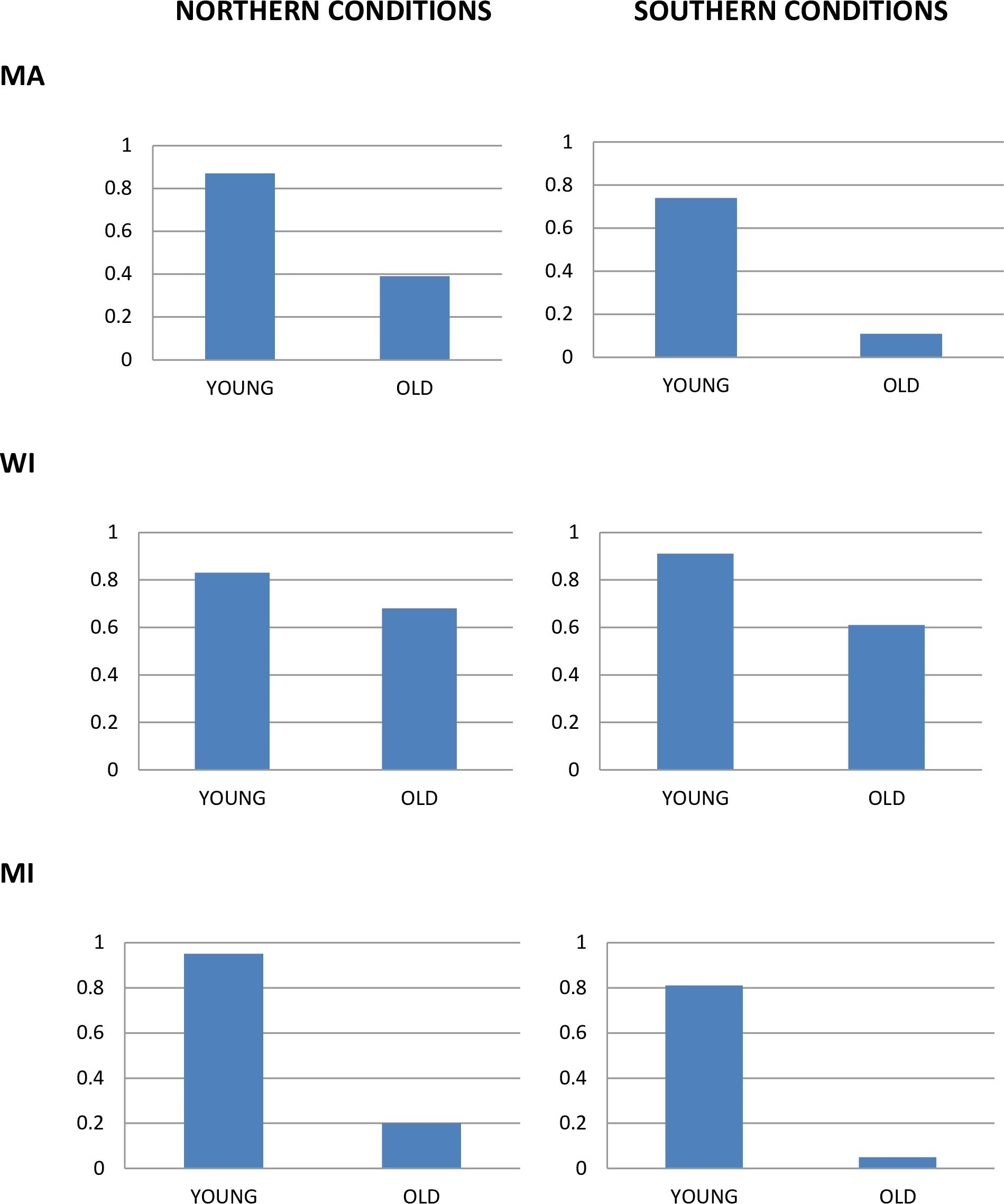

Supplement: S1 Fig — Mean proportion of larvae alive at end of experiments (672 hrs) for larvae from same cohorts that were tested twice at different ages. These were larvae whose survival was compared with those of southern populations under northern and southern conditions [12]. Larvae from northern and southern populations often did not emerge simultaneously, so larvae from MA, WI, and MI were tested twice, once when the larvae from the southern sites were tested, and later when the larvae from these northern sites were the same ages as the larvae from the southern sites (those comparisons were reported by Ginsberg et al. 2014 [12]). The results shown here from MA and WI larvae indicate survival at ~95% RH, while those from MI were at ~85% RH. Approximate mean ages of the ticks were: MA and WI, young 63 days, old 108 days; MI, young 22 days, old 77 days. (TIF) [file pone.0168723.s007.tif]

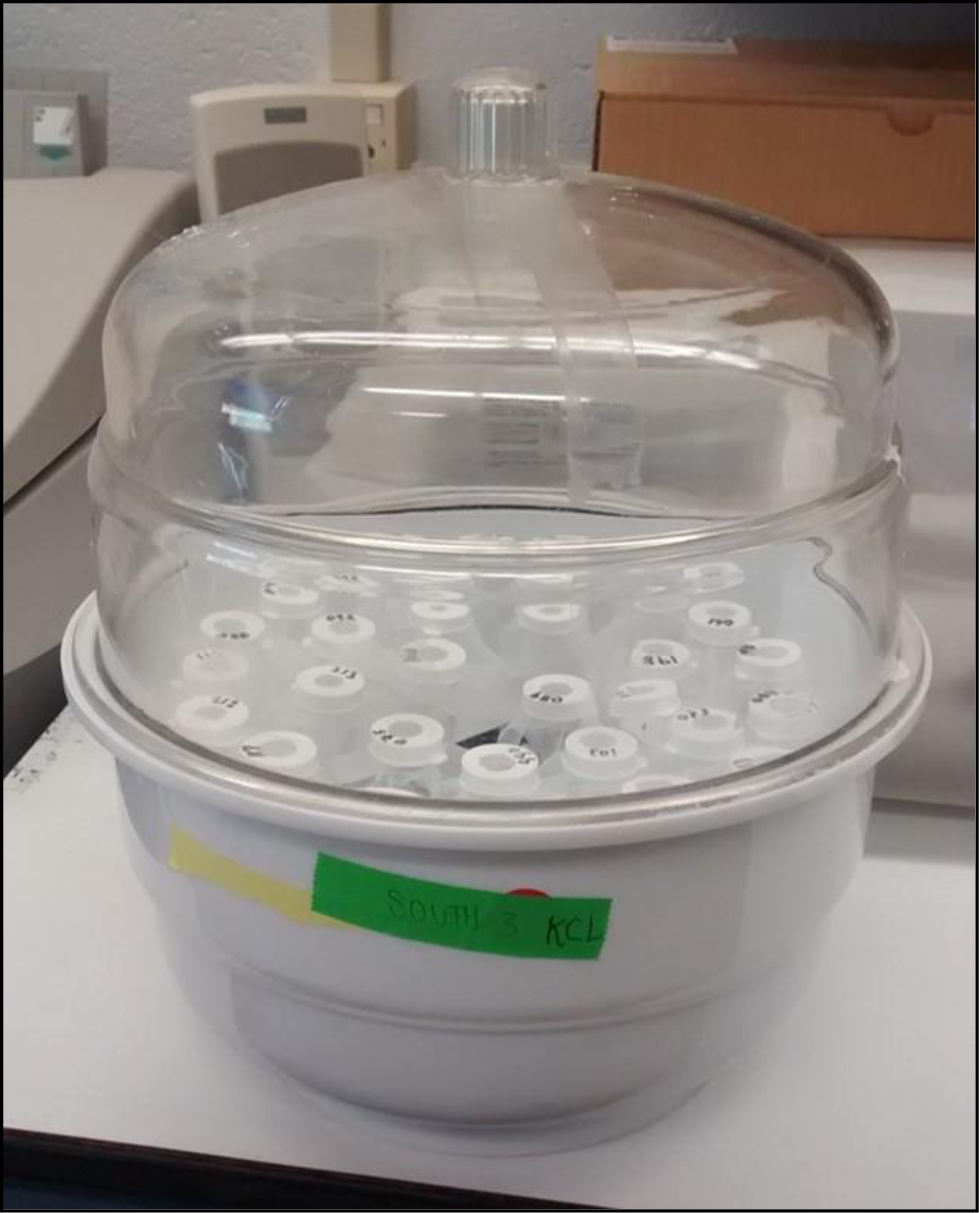

Supplement: S2 Fig — (TIF) [file pone.0168723.s008.tif]
